# Distribution of maternal red cell antibodies and the risk of severe alloimmune haemolytic disease of the foetus in a Chinese population: a cohort study on prenatal management

**DOI:** 10.1186/s12884-020-03235-w

**Published:** 2020-09-16

**Authors:** Si Li, Zhiming He, Yanmin Luo, Yanli Ji, Guangping Luo, Qun Fang, Yu Gao

**Affiliations:** 1 Department of Obstetrics, the Sixth Affiliated Hospital of Sun Yat-sen University, 510655 Guangzhou, Guangdong China; 2 Foetal Medicine Centre, Department of Obstetrics and Gynaecology, the First Affiliated Hospital of Sun Yat-sen University, Guangdong 51000 Guangzhou, China; 3 Insititute of Clinical Blood Transfusion, Guangzhou Blood Centre, 510095 Guangzhou, China

**Keywords:** alloimmunization, haemolytic disease of the foetus, Chinese, foetal anaemia, multiple antibodies

## Abstract

**Background:**

Haemolytic disease of the foetus and newborn (HDFN) is the most common aetiology of haemolytic anaemia and hyperbilirubinaemia in foetuses and neonates. Studies on the distribution of antibodies that cause haemolytic disease of the foetus (HDF) in China are limited, and the effects of multiple antibodies on the severity of HDF need further evaluation.

**Methods:**

An observational cohort study from January 2005 to December 2019 was conducted in two hospitals affiliated with Sun Yat-sen University. Maternal red cell alloimmunization was identified by the Guangzhou Blood Centre. In total, 268 pregnant woman-foetus pairs were divided into four groups according to the type of maternal alloantibodies: anti-D, anti-D combined with other antibodies, other single-antibody and other multiple antibodies. The obstetric history, antibody characteristics, incidence of severe HDF and foetal outcomes were collected and compared. Logistic regression analysis of the risk factors for HDF and survival analysis of the severe HDF-free interval were conducted.

**Results:**

Anti-D was the most common cause of HDF, followed by anti-M. No anti-K- or isolated anti-c-associated HDF was found. The incidence of severe HDF was higher in the group with anti-D combined with other antibodies than in the group with anti-D alone (*P* = 0.025), but no significant difference was found in haemoglobin level and reticulocyte count in the anaemic foetuses between these two groups. Foetuses in the other single-antibody group had a lower reticulocyte count (*P* = 0.007), more IUTs (*P* = 0.007) and an earlier onset of severe HDF (*P* = 0.012). The maximum antibody titre was significantly lower in the other single-antibody group than in the anti-D group (*P* < 0.001). A high maternal antibody titre (*P* < 0.001), multiple affected pregnancies (*P* < 0.001) and other single-antibody (*P* = 0.042) were independent risk factors for HDF. A higher reticulocyte count (*P* = 0.041) was an independent risk factor for severe HDF in anaemia foetuses affected by Rh(D) alloimmunization.

**Conclusions:**

The distribution of HDF-associated antibodies in China is different from that in Western countries. Other single non-Rh(D) antibodies could increase the risk of HDF, and anti-D combined with other antibodies would not influence the severity of foetal anaemia compared with anti-D alone.

## Background

Haemolytic disease of the foetus and newborn (HDFN) is the most common aetiology of haemolytic anaemia in foetuses and hyperbilirubinaemia in neonates[1]. As the widespread prophylactic use of anti-D immunoglobulin has greatly decreased the incidence of Rh(D) alloimmunization from 16 to 0.3% in Western countries[2, 3], the incidence of HDFN caused by non-Rh(D) antibodies has increased to 2.8‰~3.3‰[4–6]. However, the incidence of anti-D-related HDFN still contributes to the morbidity and mortality of foetuses and newborns in China[7]. A total of 60.8% of haemolytic disease is caused by anti-D, followed by anti-E, anti-c and antibodies in the MNS system, during the neonatal period[8, 9]. Thus far, the distribution of non-ABO antibodies that cause haemolytic disease of the foetus (HDF) in China is not well known. In addition, some women have multiple red cell antibodies, which might lead to a more complicated situation during pregnancy management than when a single red cell antibody is present. Some studies have found that foetuses affected by multiple antibodies need more interventions than those affected by only anti-D[10]. Thus, the objective of our study was to characterize the distribution of antibodies that cause HDF and to evaluate the effects of different antibodies on the severity of HDF in a Chinese population.

## Methods

### Study population

This was a retrospective two-centre cohort study including pregnant women with non-ABO red cell alloimmunization and their foetuses in the First Affiliated Hospital and the Sixth Affiliated Hospital of Sun Yat-sen University from January 2005 to December 2019. All patients provided written informed consent for each medical intervention. The Guangzhou Blood Centre identified the antibodies responsible for red cell alloimmunization. We screened the alloantibodies in pregnant women based on the following criteria: (1) having Rh(D) negative phenotype; (2) having previous adverse pregnancy outcomes, including recurrent abortion, foetal demise, and hydrops fetalis; (3) identification of unexpected alloantibodies in ABO blood typing; and (4) having a previous history of blood transfusion. The commercial panel of reagent cells (Immucor, Norcross, GA, USA) with the saline tube test and an indirect antiglobulin test (IAT) with the DG Gel Coombs card (GRIFOLS, Barcelona, Spain) were used for antibody screening and identification. When immunoglobin M (IgM) alloantibody was detected, DTT (0.01 mol/L) was used to destroy the IgM antibody first and then IgG alloantibody was determined by IAT using the tube method. The titre of IgG was determined by the IAT method using the reagent cells in the DG Gel Coombs card (GRIFOLS, Barcelona, Spain) after incubation at 37 °C for 15 min. We included women with IgG red cell antibodies. For the women both had IgM and IgG, only the titre of IgG was recorded in this study. The antenatal diagnostic criteria for HDF were as follows: (1) the detection of non-ABO IgG antibodies in the maternal serum and corresponding maternal-foetal blood group incompatibility; (2) foetal anaemia confirmed by cordocentesis or an adverse pregnancy outcome, which included hydrops fetalis and foetal demise; and (3) a positive antibody elution test from foetal red blood cells (RBCs), which was direct evidence of causing haemolysis. In cases of missing values, treatment with intrauterine transfusion (IUT), hydrops fetalis and foetal demise were also considered as valid confirmation of clinically relevant HDF. We excluded women and their foetuses based on the following criteria: (1) the woman underwent termination of the pregnancy due to foetal structure or chromosome abnormalities; (2) a woman with alloimmunization had the same blood group phenotype as that of her foetus; (3) the woman had unknown antibodies; or (4) the woman was lost to follow-up. Stillbirth cases in this study did not include foetal death due to the discontinuation of treatment and induction of labour. Not all the data were available for each case.

### Data collection

Data on the maternal obstetric history, blood transfusion history, type of alloimmunization, presence or absence of hydrops fetalis, and foetal sex were collected. For the anaemic foetuses, we further collected data on the gestational age at the time of diagnosis of HDF; foetal haemoglobin levels, haematocrit levels, reticulocyte counts, and reticulocyte percent before IUT; the number of IUTs; and foetal outcomes. Women who underwent antibody detection multiple times during the same pregnancy were included as a single entry, and the highest titre was recorded during the whole pregnancy. For the women with multiple antibodies, we recorded the titres of all types of antibodies and used the highest titre into analysis. Regarding the women who were pregnant more than once during the study period, each pregnancy was included in our report. To determine the effects of different alloimmunizations, the patients were classified into four groups according to their antibodies, namely, only anti-D (anti-D group), anti-D combined with other antibodies (anti-D combined with others group), other single-antibody (other single-antibody group) and other multiple antibodies (other multiple antibodies group).

### Definition and treatment policy

A previously affected history was defined by the existence of a previous perinatal loss related to HDFN, a previous need for IUT, or a previous need for neonatal exchange transfusion[11]. Foetal outcomes were defined as the survival of the foetuses and the gestational age at birth.

Foetal anaemia was confirmed by cordocentesis, and the severity of foetal anaemia was categorized based on the haemoglobin concentration expressed as a multiple of the median (MoM) for gestational age as follows: mild (0.83 − 0.65 MoM), moderate (0.64 − 0.55 MoM), and severe (< 0.55 MoM)[12]. The indication for an IUT was a foetal haematocrit less than 30% [11].

### Primary and secondary outcomes

The primary outcome was the occurrence of severe HDF, which was defined as severe foetal anaemia, hydrops foetalis, stillbirth, or the need for IUT due to maternal alloimmunization[1]. The secondary outcomes were the number of IUTs, the severity of foetal anaemia and the outcomes of the foetuses.

### Statistics analysis

Statistical analysis was performed using SPSS 22.0 statistical software. Quantitative variables are expressed as the medians with the 25th and 75th quartiles or the means with their standard deviations. Chi-square tests or Fisher’s exact tests were used for distribution-based comparisons between groups. The t-test was used when the values were normally distributed, while the Mann-Whitney U test was applied when the values were nonnormally distributed. The potential risk factors for HDF in foetuses with maternal alloimmunization, including previously affected pregnancies per woman, maternal transfusion history, maternal antibody titre, maternal-foetal major ABO incompatibility and the types of maternal antibodies were analysed by logistic regression analysis. The potential risk factors for severe HDF in foetuses with Rh(D) alloimmunization, including foetal sex, maternal antibody titre, gestational age at diagnosis, reticulocyte count and combination of maternal anti-D with other antibodies, were also included in the multiple variable analysis. The results are presented as p values, and a two-sided *p* value < 0.05 was regarded as statistically significant. The odds ratios (ORs) with 95% confidence intervals (95% CIs) are also presented in regression analysis. The Kaplan-Meier survival analysis of the survival time free from severe HDF is presented. To determine the short-term differences in this interval between groups, the Breslow method was used. The survival curve was generated by GraphPad Prism 5.0.

## Results

**Fig. 1:**
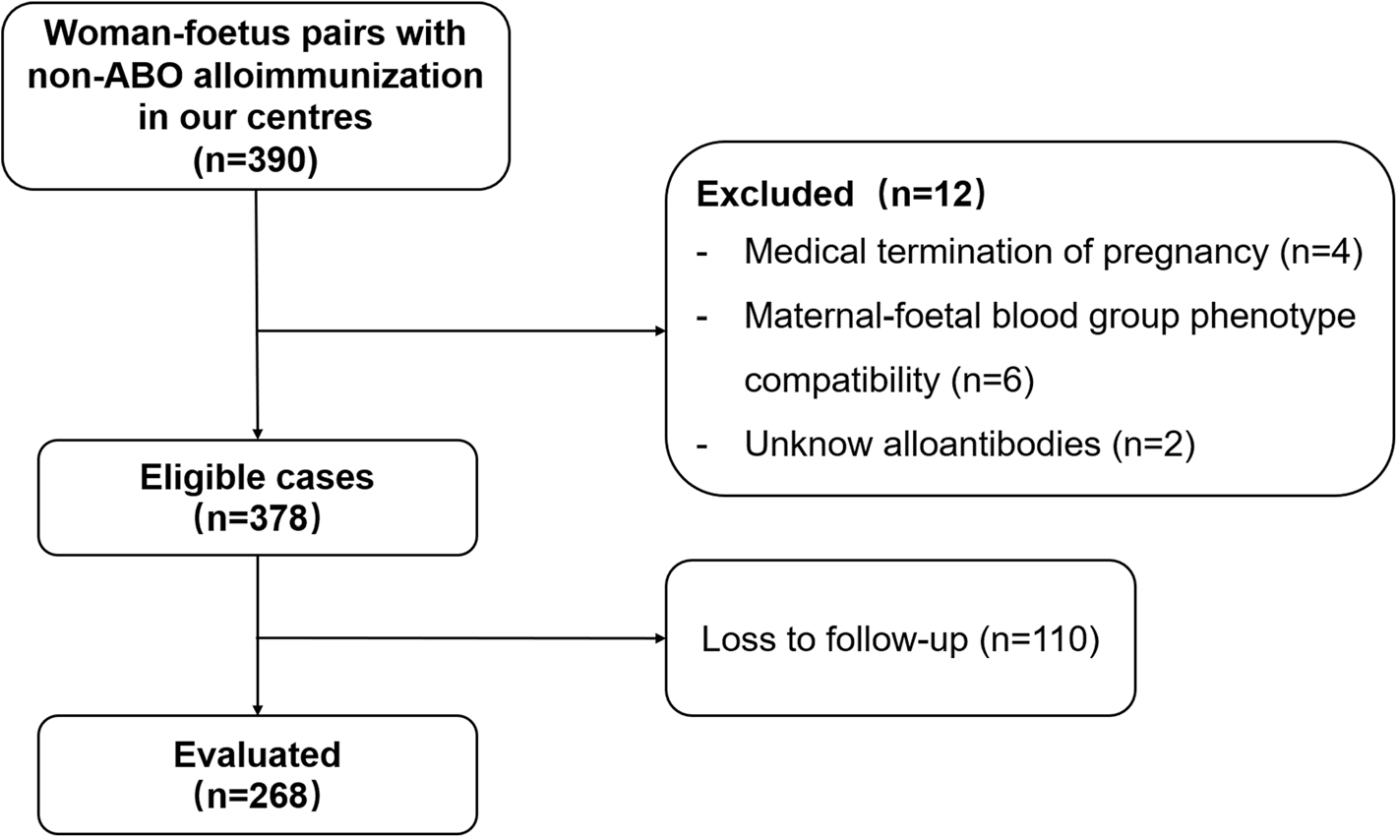
Flow chart of the study participants

From January 2005 to December 2019, a total of 390 pregnant women were positive for alloantibodies. In total, 122 cases were excluded from our study based on the following criteria: loss to follow-up (*n* = 110), termination of pregnancy due to foetal structural abnormalities (*n* = 04), specific maternal-foetal blood phenotype compatibility (*n* = 06) or unknown antibodies (*n* = 02) (Fig. 1). A total of 268 pregnant women and their foetuses were finally included in our study.

### Basic characteristics

The average age of the included pregnant women was 31.1 ± 4.3 years old (the ages of 11 women were unknown). Totally 15 women had more than one pregnancy during the study period and thus were represented more than once in our dataset. The patients in this cohort were divided as follows: anti-D group (*n* = 203), anti-D combined with other antibodies group (*n* = 38), other single-antibody group (*n* = 24) and other multiple antibodies group (*n* = 03). In the anti-D combined with other antibodies group, there were 28 women with anti-C, 9 with anti-E and 1 with anti-M. The anti-D group had the most women with a history of blood transfusion. The basic information of the four groups is shown in Table 1. The women in the anti-D combined with other antibodies group had significantly more gravidities (*P* = 0.002) and a higher rate of previous affected history of HDFN (*P* = 0.011) than the anti-D group (Table 1).

**Table 1:** Baseline data stratified by group

	Anti-D *n* = 203	Anti-D combined with others *n* = 38	Other single-antibody *n* = 24	Other multiple antibodies *n* = 03^†^
Maternal age (years)	31.5 ± 4.3 (Unknown = 11)	30.5 ± 4.5	29.3 ± 4.2	29.0 ± 3.6
*P*		0.368	0.125	-
Gravidity (times)	3 (2–4) (Unknown = 9)	4 (3–5)^a^	3 (2-3.8)	5 (2.1-5)
*P*		**0.002**	0.262	-
1	8	1	3	0
2	53	4	6	1
>2	133	33	15	2
Maternal transfusion history *n*(%)	30 (14.8)	7 (18.4)	2 (8.3)	1 (-)
*P*		0.568	0.543	-
Women with previously affected history *n*(%)	80 (40.8) (Unknown = 7)	24 (63.2)^a^	14 (58.3)	2 (-)
*P*		**0.011**	0.075	-
Maternal-foetal ABO incompatibility *n*(%)	27 (14.2) (Unknown = 13)	4 (10.5)	4 (20.0) (Unknown = 4)	0 (-)
*P*		0.545	0.507	-
Foetal sex				
Female *n*	89	18	6	1
Male *n*	100	20	15	1
Unknown *n*	14	0	3	1

^a^Significant difference compared to the anti-D group;

^†^Can’t calculate the *p* value due to the small sample size in this group

### Incidence of HDF among cases with different types of antibodies

There were 45.5% (122/268) of the foetuses suffering from HDF, and 34.7% (93/268) of them received IUTs. Anti-D was the most common antibody (82/122, 67.2%) causing HDF, and 78.0% (64/82) of patients underwent IUTs (Table 2). The cases of other single-antibody included anti-M (*n* = 18), anti-E (*n* = 03) and anti-Mur (*n* = 03). The cases of multiple antibodies included anti-Ec (*n* = 02) and anti-Ce (*n* = 01). The anti-M antibody in the MNS system was the second most common antibody outside of those in the Rh system.

**Table 2:** The incidence of severe HDF between different types of antibodies

	Anti-D *n* = 203	Anti-D combined with others *n* = 38	Other single-antibody *n* = 24	Other multiple antibodies^§^ *n* = 03
HDF *n*(%)	82 (40.4)	23 (60.5)^b^	15 (62.5)^b^	2 (-)^c^
*P*		**0.022**	**0.038**	-
Severe HDF^a^*n*(%)	73 (36.0)	21 (55.3)^b^	15 (62.5)^b^	2 (-)^c^
*P*		**0.025**	**0.012**	-
Severe anaemia *n*(%)	38 (18.7)	9 (23.7)	8 (33.3)	0
*P*		0.478	0.307	-
Hydrops fetalis *n*(%)	18 (8.9)	3 (7.9)	8 (33.3)^b^	0
*P*		> 0.999	**0.002**	-
Intrauterine demise *n*(%)	12 (5.9)	2 (5.3)	3 (12.5)	1 (-)^c^
*P*		> 0.999	0.202	-
IUT *n*(%)	64(31.5)	18 (47.4)	10 (41.6)	1 (-)^c^
*P*		0.059	0.316	-
Survival rate of HDF				
* Foetuses*	55 (67.1)	18 (78.3)	12 (80.0)^b^	1 (-)^c^
* P*		0.303	0.380	-
* Neonates*	55 (67.1)	18 (78.3)	9 (60.0)	1 (-)^c^
* P*		0.303	0.595	-

*HDF* haemolytic disease of the foetus; *IUT* intrauterine transfusion

^a^Foetuses may fall into more than 1 severe HDF defining category

^b^Significant difference compared to the anti-D group

^c^Can’t calculate the percentage because the denominator was less than 20

^§^Can’t calculate the *p* value due to the small sample size in this group

Furthermore, different types of antibodies were associated with various risks of HDF. The incidence of severe HDF in the anti-D combined with other antibodies group was significantly higher than that in the anti-D group (55.3% vs. 36.0%, *P* = 0.025). The need for IUT in the anti-D combined with other antibodies group tended to be higher than that in the anti-D group (47.4% vs. 31.5%, *P* = 0.059). The other single-antibody group had a significantly higher incidence of HDF (62.5% vs. 40.4%, *P* = 0.038), hydrops fetalis (33.3% vs. 8.90%, *P* = 0.002) and severe HDF (62.5% vs. 36.0%, *P* = 0.012) than the anti-D group (Table 2). The anti-M-associated risk of severe HDF (10/18, 55.6%) was not significantly different from the anti-D-associated risk (73/203, 36.0%, *P* = 0.100). In 41 women with multiple antibodies, the red cell antigens of ABO blood group in 40 foetuses and Rh(D) antigens were known in all foetuses during the foetal or neonatal period, but only 21 of them had further identified other antigens (C and c, E and e) in the Rh system and 1 case had further identified the M and N antigen. All these women in the anti-D combined others group had foetuses with positive Rh(D) antigen, 65.0% (13/20) of them had other cognate antigens. In the other multiple antibodies group, 2 foetuses those who had the same mother, had cognate antigens in Rh system.

There were 17 cases of intrauterine demise, including 11 due to anti-D, 2 due to anti-D with anti-C, 2 due to anti-M, 1 due to anti-Mur and 1 due to anti-Ec. Nine of these 17 foetuses did not survive after IUT. Demise in the other 8 cases was due to discontinuation of treatment and termination of the pregnancy because of severe foetal hydrops or severe foetal anaemia. Three foetuses were delivered by emergency caesarean but died during the neonatal period. The survival rate of HDF was not different among the 4 groups.

### Risk factors for HDF in foetuses with maternal alloimmunization

As there were only 3 cases in the other multiple antibodies group, we did not include them into analysis. In the univariable analysis among the foetuses with maternal alloimmunization, four variables were associated with the occurrence of HDF (Table 3): more previous affected pregnancies per woman (OR 5.66, CI 3.561–9.006), a higher maternal antibody titre (OR 1.00, CI 1.001–1.002), anti-D combined with others (compared with anti-D, OR 2.26, CI 1.114–4.594), and other single-antibody (compared with anti-D, OR 2.46, CI 1.028–5.886). There were no significant differences between HDF and non-HDF foetuses with regard to maternal blood transfusion history or major ABO incompatibility. In the multivariable analysis, we included all seven variables. There were four variables associated with the occurrence of HDF: more previous affected pregnancies (OR 5.34, CI 3.156–9.036), a maternal transfusion history (OR 3.74 CI 1.219–11.464), a higher maternal antibody titre (OR 1.00, CI 1.001–1.002), and other single-antibody (compared with anti-D, OR 3.25, CI 1.043–10.114).

**Table 3:** Risk factors for HDF in foetuses with maternal alloimmunization

	HDF (*n *= 120)	No HDF (*n* = 145)	*P*	Univariable OR (95% CI)	*P*	Multivariable OR^b^ (95% CI)
Previous affected pregnancies per woman^a^	1(0–2)	0 (0–0)	**<0.001**	5.66(3.561–9.006)	**<0.001**	5.34(3.156–9.036)
Maternal transfusion history, n (%)	18(15.0)	21(14.5)	0.906	0.96(0.485–1.898)	**0.021**	3.74(1.219–11.464)
Maternal antibody titre^a^	1:512(1:256-1:1792)	1:64(1:16 − 1:256)^c^	**<0.001**	1.00(1.001–1.002)	**<0.001**	1.00(1.001–1.002)
Major ABO incompatibility, n (%)	9(7.7)^d^	26(19.8)^e^	0.319	0.72(0.376–1.375)	0.488	0.73(0.303–1.767)
Types of maternal antibody						
Anti-D, n (%)	82(68.3)	121(83.4)	**-**	-	-	-
Anti-D combined with others, n (%)	23(19.1)	15(10.3)	**0.024**	2.26(1.114–4.594)	0.598	0.74(0.246–2.246)
Other single-antibody, n (%)	15(12.5)	9(4.8)	**0.043**	2.46(1.028–5.886)	**0.042**	3.25(1.043–10.114)

*HDF* haemolytic disease of the foetus

^a^Data present as median (25th − 75th quartiles)

^b^The multivariable analysis included the following risk factors for analysis: previous affected pregnancies per woman, maternal transfusion history, maternal antibody titre, major ABO incompatibility and types of maternal antibody (anti-D, other single-antibody)

^c^Assessed in 135/145 (93.1%) foetuses, missing values for 10

^d^Assessed in 117/120 (97.5%) foetuses, missing values for 3

^e^Assessed in 131/145 (90.3%) foetuses, missing values for 14

### Foetal anaemia among groups with different types of antibodies

**Fig. 2:**
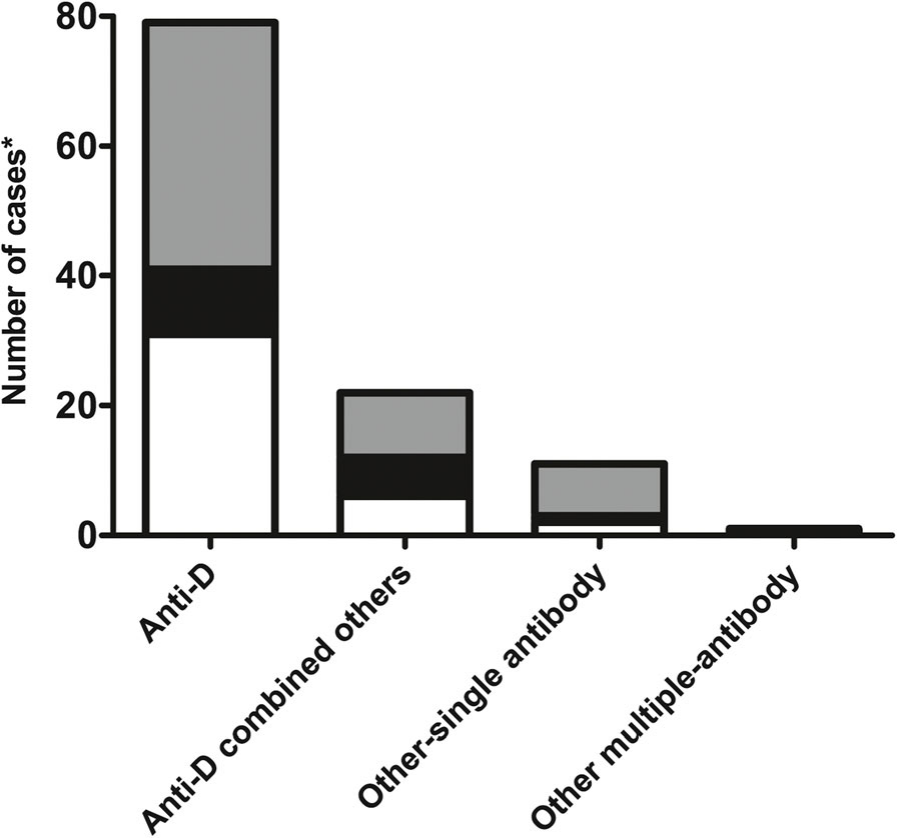
The distribution of antibodies that resulted in foetal haemolytic anaemia; White: mild anaemia; Black: moderate anaemia; Gray: severe anaemia. *There were six cases of foetal demise before cordocentesis (3 with anti-D, 2 with anti-M and 1 with anti-Ec). The pregnancies were terminated in three cases (1 with anti-DC ,1 with anti-Mur and 1 with anti-M) without cordocentesis

Among the foetuses suffering from HDF, there were six cases of foetal demise before cordocentesis (3 with anti-D, 2 with anti-M and 1 with anti-Ec). The pregnancies were terminated in two cases (1 with anti-Mur and 1 with anti-M) because of severe foetal hydrops without cordocentesis. One foetus (anti-DC-related HDF) was delivered via emergency caesarean at 34^+ 5^ weeks of gestation because of foetal distress and a rapid increase in the antibody titre. Therefore, a total of 113 foetuses with haemolytic anaemia confirmed by cordocentesis were included in the analysis. The distribution of antibodies that resulted in foetal haemolytic anaemia is shown in Fig. 2. There was no significant difference in the distribution of the degree of foetal anaemia among the four groups (*P* = 0.466). The antibodies in the Rh blood group system were the most common antibodies leading to foetal anaemia (92.0%, 104/113), followed by the antibodies in the MNS blood group system, including anti-M (*n* = 07) and anti-Mur (*n* = 02). Anti-D was the most common antibody that caused foetal anaemia. It was effective alone (79/113, 69.9%) or in combination with anti-C (15 cases) or anti-E (7 cases) (22/113, 19.5%) with regard to causing foetal haemolytic anaemia. Anti-E caused one case of severe anaemia, one case of mild anaemia and one case of moderate anaemia with anti-c.

Regarding the characteristics of foetuses with anaemia and their mothers, those who had anti-D combined with other antibodies had more pregnancies affected by HDFN than those who had anti-D alone [1 (0–1) vs. 2 (1–2), *P* = 0.002]. However, there was no significant difference in the foetal haemoglobin concentration, haematocrit, reticulocyte count and percentage, or gestational age at birth between these two groups (Table 4). The maximal antibody titre was significantly lower in the other single-antibody group [1:32 (1:8 − 1:256)], as well as that of anti-M [1:8 (1:8 − 1:64)], than those in the anti-D group [1:512 (1:512-1:2048)] (*P <* 0.05). In addition, the foetuses in the other single-antibody group had a significantly lower reticulocyte count (142.1 ± 122.6 × 10^9^/L vs. 303.0 ± 97.3 × 10^9^/L, *P* = 0.007) and reticulocyte percentage [8.5 (4.2–17.1)% vs. 15.7 (10.1–22.8)%, *P* = 0.015] but a higher number of IUTs [5 (3–5) vs. 2 (1–4), *P* = 0.007] than those in the anti-D group (Table 4).

**Table 4:** Characteristics of anaemic foetuses in the three groups

	Anti-D *n* = 79	Anti-D combined with others *n* = 22	*P*	Other single-antibody *n* = 11	*P*
Previously affected pregnancies per women	1(0–1)	2(1–2)	0.002^*^	1(0–1)	0.990
Maternal antibody titre	1:512 (1:512-1:2048)	1:768 (1:256-1:2560)	0.561	1:32 (1:8 − 1:256)	<0.001^*^
Haemoglobin (g/L)^a^	68.4 ± 26.6	67.7 ± 23.6	0.908	50.2 ± 23.1	0.033^*^
Haematocrit (%)^a^	20.9 ± 7.7	20.5 ± 6.3	0.831	15.2 ± 6.6	0.023^*^
Reticulocyte count (× 10^9/L)^a^	303.0 ± 97.3^c^	331.3 ± 104.9	0.245	142.1 ± 122.6^d^	0.007^*^
Reticulocyte percentage (%)^b^	15.7 (10.2–22.8)^c^	19.9(13.6–31.3)	0.168	8.5 (4.2–17.1)^d^	0.015^*^
Hydrops, *n* (%)	15(19.0)	3 (13.6)	0.756	3 (33.3)	0.235
Number of IUTs^c^	2(1–4)	3.5 (1–5)	0.167	5 (3–5)	0.007^*^
Gestational age at birth^b,^ ^c^	35.0 (33.9–36.6)	36.0 (33.4–36.9)	0.586	35.0(33.2–35.6)	0.331

*IUT* intrauterine transfusion

*Significant difference compared to the anti-D group

^a^Data presented as the mean ± SD

^b^Data present as median (25th − 75th quartiles)

^c^Assessed in 72/79 (91.1%) foetuses, missing values for 7

^d^Assessed in 6/9 (66.7%) foetuses, missing value for 3

### Risk factors for severe HDF in foetuses with Rh(D) alloimmunization

In the univariable analysis among the foetuses affected by anti-D, including those combined with other antibodies, we included five variables: foetal sex, maternal titre, diagnostic gestational age, foetal reticulocyte count and ani-D combined with other antibodies in the analysis (Table 5). Two variables were significantly associated with the occurrence of severe HDF: an early diagnostic gestation age of HDF (OR 0.71; CI 0.544–0.934) and a higher reticulocyte count (OR 1.01; CI 1.003–1.022). In the multivariable analysis, only one variable was associated with the occurrence of severe HDF: a higher reticulocyte count (OR 1.01; CI 1.001–1.026). The foetal sex, maternal antibody titre, diagnostic gestation age and anti-D combined with other antibodies in mothers were not the independent risk factors for severe HDF.

**Table 5:** The predictors of severe HDF in foetuses with anti-D and/or combined other antibodies

	Severe HDF (*n* = 91)	No severe HDF (*n* = 10)	*P*	Univariable OR (95% CI)	*P*	Multivariable OR^c^ (95% CI)
Female, n *(*%)	42 (46.2)	7 (70.0)	0.446	0.60 (0.157–2.257)	0.067	0.17 (0.026–1.138)
Maternal antibody titre^a^	1:1024 (1:512-1:2048)	1:320 (1: 56 − 1:1024)	0.067	1.00 (1.000-1.003)	0.671	1.00 (0.999–1.002)
Gestational age at diagnosis, weeks^a^	28.0 (23.3–32.1)	33.6 (32.7–34.0)	**0.014**	0.71 (0.544–0.934)	0.098	0.76 (0.543–1.053)
Reticulocyte count^b^ (10^9^/L)	318.4 ± 98.8	226.7 ± 59.4	**0.012**	1.01 (1.003–1.022)	**0.041**	1.01 (1.001–1.026)
Anti-D combined with others, n (%)	21 (23.1)	1 (10.0)	0.359	2.70 (0.323–22.556)	0.834	1.29 (0.119–14.052)

*HDF* haemolytic disease of the foetus; *OR* odds ratio

^a^Data present as the median (25th − 75th quartiles)

^b^Data presented as the mean ± SD

^c^The multivariable analysis included the following risk factors for analysis: foetal sex, maternal antibody titre, gestational age at diagnosis, reticulocyte count and anti-D combined with others

### Severe HDF-free survival

**Fig. 3:**
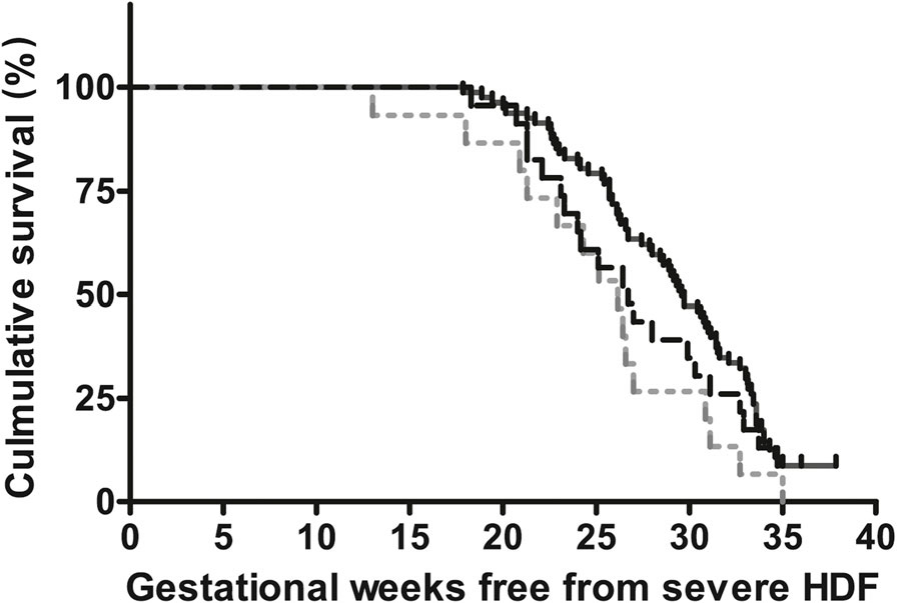
Kaplan-Meier curve of the gestational age interval free from severe HDF between groups; HDF: haemolytic disease of the foetus;——: anti-D; ---: anti-D combined with others; ……: Other single-antibody

Totally 45.5% (122/268) of the foetuses in our study were affected by maternal alloimmunization and developed HDF. We performed a survival analysis with the Kaplan-Meier method to determine the gestational age interval free from severe HDF (Fig. 3). As the only 2 foetuses in the other multiple antibodies group were from the same pregnant woman, we did not include them in the analysis. The median survival times free from severe HDF were significantly different among the three groups (*P* = 0.028). In the anti-D group, 11.0% of foetuses did not develop severe HDF (Table 2). The median survival time free from severe HDF was 29.6 weeks, and the longest survival time free from severe HDF was 37.9 weeks in this group. When compared to the anti-D combined with other antibodies group, the median survival time was not significantly different (29.6 weeks vs. 26.7 weeks, *P* = 0.128). However, the median survival time free from severe HDF in the other single-antibody group was significantly shorter than that in the anti-D group (26.2 weeks vs. 29.6 weeks, *P* = 0.012), suggesting that the foetuses in the other single-antibody group might be affected by maternal alloimmunization earlier.

## Discussion

The distribution of maternal alloimmunization and HDFN varies in different countries and populations[7, 13]. Thus far, the distribution of HDF antibodies in the Chinese population has not been reported. In Guangdong Province of China, the percentage of pregnant women with red cell alloimmunization was 0.27%~1.05%[14]. In our study, we analysed 268 pregnant women with red cell alloimmunization and their foetuses, including 122 foetuses with haemolytic disease, to characterize the antibody distribution of HDF in a Chinese population. Anti-D was still the most common cause of HDF (67.2%, 82/122). However, in our study, no case of HDF due to isolated anti-c was found, which is the secondary cause of severe HDF after anti-D in the Caucasian population[15]. The difference might be due to the significantly lower frequency of the c antigen in the Chinese population than in the Caucasian population[16]. Similarly, there was no case of anti-K HDF detected in our study, nor were there any women who were positive for the anti-K antibody. Due to the extremely low frequency of the K antigen in the Kell blood group system in the Chinese population, anti-K antibody positivity is extremely rare[7], and anti-K-related HDN has rarely been reported[17]. Therefore, the distribution of HDF-associated antibodies in the Chinese population is different from that in the Caucasian population.

Moreover, we found that anti-M was the most common non-Rh(D) antibody, accounting for 55.5% of the incidence of HDF (10/18). Very few reports have causally linked anti-M antibody positivity with HDFN in a Caucasian population[18]. Over the past two decades, anti-M-related HDFN has mostly been reported in Asian ethnic groups, especially in Japanese[19] and Chinese populations[20, 21], and it has been reported to result in severe foetal anaemia, hydrops fetalis, stillbirth and neonatal death. A previous study found that 88.6% of anti-M-related HDFN cases occurred in Asian populations[21], indicating the high pathogenicity of IgG anti-M in Chinese people. Even though our tertiary prenatal centre provided a relatively higher incidence of HDF due to anti-M, our results suggested that the risk of anti-M-associated severe HDFN in the Chinese population might be high, with 60.0% (6/10) receiving IUTs and 2 cases of intrauterine demise due to the lack of timely treatment. In addition, the antibody titre of anti-M was significantly lower than that of anti-D, indicating that anti-M can cause severe HDF even at low titres. The lower reticulocyte count indicated incompatible erythropoiesis with severe anaemia [21], which was also found in neonatal cases[19]. These results might be explained by the suppression of red cell development from erythroid precursor cells [22, 23]. As a consequence, anti-M-related HDFN can have a negative result of direct anti-human globulin test (direct Coombs test) [19], making the diagnosis of anti-M-related HDFN difficultly and often missed.

The presence of multiple antibodies seemed to increase the risk of HDF-associated morbidity without influencing the severity of foetal anaemia. The incidence of severe HDF was significantly higher when the pregnant women had anti-D combined with other antibodies, which was consistent with previous studies [24–26]. However, after the logistic regression analysis, anti-D combined with other antibodies was not an independent risk factor for HDF. Moreover, among the anaemic foetuses, no significant difference was found in the foetal haemoglobin concentration or gestational age at diagnosis between the anti-D group and the anti-D combined with other antibodies group. The similar reticulocyte counts and percentages between these two groups indicated similar haematopoietic conditions. Some studies reported that neither the foetal anaemia severity nor the gestational age at first IUT was influenced by anti-D with other antibodies[10, 27]. In the logistic regression analysis of anaemic foetuses affected by Rh(D) alloimmunization, anti-D combined with other antibodies was not a risk factor for severe HDF in either univariate analysis or multivariate analysis. These results indicated that the high incidence of HDF in the anti-D combined with other antibodies group was affected by other confounding factors. Furthermore, we also found that women with multiple antibodies had a higher rate of previous HDFN and more affected pregnancies than those with only anti-D. These results might suggest that the more often an Rh(D)-negative woman is exposed to an Rh(D)-incompatible foetus, the higher chances of generating additional antibodies are, leading to a more aggressive immune response and cumulative effect and increasing the risk of haemolysis and the chance of developing severe HDF. For the foetuses affected by maternal multiple antigens, 68.2% of the foetuses, those who identified other Rh blood group phenotypes, had cognate antigens. The additional antibody of the remaining cases might occur from previously affected pregnancy or the stimulation from nature. These further indicated as anti-D still played a dominant role in haemolysis, the severity of HDF was not significantly different regardless of whether anti-D was present alone or in combination with other antibodies. Therefore, when a pregnant woman has multiple antibodies and a history of multiple HDFN affected pregnancies, her foetus might have a higher risk of developing severe HDF and should be closely monitored during the antenatal period.

In the regression analysis, we found that a high maternal antibody titre, more previously affected pregnancies, and other single-antibody were independent risk factors for the occurrence of HDF. Even though in some alloimmunizations, a low antibody titre can cause severe HDFN[28], a high titre suggests a more active immune response, increasing the risk of HDF. Moreover, a history of multiple affected pregnancies might result in the generation of a larger amount and longer duration of antibodies [29], which can cross the placenta to cause the disease in the foetal period. In Rh(D) alloimmunization, once foetal anaemia occurred, the antibody titre could not predict the risk of severe HDF. A higher reticulocyte count was an independent risk factor for severe HDF. The more severe the anaemia and the more active the erythropoiesis were, the more severe the disease, indicating the need for intrauterine intervention. In the multivariable analysis, we also found that a transfusion history in the mother might be associated with HDF. According to the Technical Specifications of Clinical Blood Transfusion in China, no blood types other than ABO and Rh(D) are regularly detected before blood transfusion. Due to the scarcity of Rh(D) negative blood in China, Rh(D) negative individuals might receive Rh(D) positive red blood cells in some medical emergencies. As the volume of the blood transfusion is larger than the volume of foetomaternal haemorrhage and is enough to sensitize the individual[29], sensitized women would develop more aggressive alloimmunization during pregnancy. Therefore, women with a previous blood transfusion history might have a higher risk of having a foetus with HDF.

The Kaplan-Meier analysis was conducted to determine the severe HDF-free interval and compare the intervals among groups with different antibodies, and the results provided a clinical reference to support the estimation of the gestational age at the onset of severe HDF and the appropriate time for clinical intervention. The significant difference in the median survival time among the groups indicated that the onset time of severe HDF varied based on the types of antibodies. The other single-antibody group had a significantly shorter survival time, providing evidence that foetuses affected by other single-antibody group, mainly anti-M, might develop severe HDF earlier than those affected by anti-D alone. This can be explained by the earlier development of antigens in the MNS system than in the Rh system during foetal development and erythropoiesis[30].

There were still some limitations of our study. Our centre is a tertiary prenatal care centre, and the patients therein might have a higher risk for HDFN, more severe HDFN, and more severe associated complications. Therefore, the morbidity and mortality rates might be higher among patients in this study than among individuals throughout the country. Because of the insufficient sample size for specific non-anti-D antibodies, we could not compare the differences between specific antibodies, especially in terms of multiple other antibodies. As the inadequate realization of the maternal alloimmunization with multiple antibodies in the early stage of our centre, not all the foetuses were identified the non-Rh(D) blood phenotype, and gave a limited analysis for multiple maternal antibodies and foetal cognate antigens. What’s more, the survival curves between the anti-D and anti-D combined with other antibodies groups intersected, indicating that confounding factors might have affected the statistical results. As the period free from severe HDF can be influenced by the frequency of foetal investigations, gestational age at the time of prenatal consultation, and awareness of the disease, which varied over the 15-year period, the results of the Kaplan-Meier analysis may not be accurate for all antibody patterns. More prospective studies are needed for further accurate investigation on different alloimmunizations.

## Conclusions

The antibody distribution for HDF in China is different from that in Western countries. Other single non-Rh(D) antibodies could increase the risk of HDF, and anti-D combined with other antibodies is not an independent risk factor of HDF and would not influence the severity of foetal anaemia compared with anti-D alone.

## Data Availability

The datasets used and/or analysed during the current study are available from the corresponding author on reasonable request.

## Abbreviations

CI: Confidence interval
HDF: Haemolytic disease of the foetus
HDFN: Haemolytic disease of the foetus and new-born
IAT: Indirect antiglobulin test
IgG: Immunoglobin G
IgM: Immunoglobin M
IUT: Intrauterine transfusion
MoM: Multiple of the median
OR: Odds ratio
RBC: Red blood cell
